# *Mobile Link* – a theory-based messaging intervention for improving sexual and reproductive health of female entertainment workers in Cambodia: study protocol of a randomized controlled trial

**DOI:** 10.1186/s13063-018-2614-7

**Published:** 2018-04-19

**Authors:** Carinne Brody, Sovannary Tuot, Pheak Chhoun, Dallas Swendenman, Kathryn C. Kaplan, Siyan Yi

**Affiliations:** 1 Public Health Program, College of Education and Health Sciences, Touro University California, Vallejo, CA USA; 2 KHANA Center for Population Health Research, No. 33, Street 71, Phnom Penh, Cambodia; 3 Department of Psychiatry and Biobehavioral Sciences, University of California, Los Angeles, CA USA

**Keywords:** Female entertainment workers, HIV, mHealth, Mobile Link, Sexual and reproductive health, Randomized controlled trial, Study protocol, Cambodia

## Abstract

**Background:**

In Cambodia, HIV prevalence is concentrated in key populations including among female entertainment workers (FEWs) who may engage in direct or indirect sex work. Reaching FEWs with sexual and reproductive health (SRH) services has been difficult because of their hidden and stigmatized nature. Mobile-phone-based interventions may be an effective way to reach this population and connect them with the existing services. This article describes study design and implementation of a randomized controlled trial (RCT) of a mobile health intervention (the *Mobile Link*) aiming to improve SRH and related outcomes among FEWs in Cambodia.

**Methods:**

A two-arm RCT will be used to determine the effectiveness of a mobile-phone-based text/voice messaging intervention. The intervention will be developed through a participatory process. Focus group discussions and in-depth interviews have been conducted to inform and tailor behavior change theory-based text and voice messages. During the implementation phase, 600 FEWs will be recruited and randomly assigned into one of the two arms: (1) a control group and (2) a mobile phone message group (either text messages [SMS] or voice messages [VM], a delivery method chosen by participants). Participants in the control group will also receive a weekly monitoring survey, which will provide real-time information to implementing partners to streamline outreach efforts and be able to quickly identify geographic trends. The primary outcome measures will include self-reported HIV and sexually transmitted infections (STI) testing and treatment, condom use, contraceptive use, and gender-based violence (GBV).

**Discussion:**

If the *Mobile Link* trial is successful, participants will report an increase in condom use, linkages to screening and treatment for HIV and STI, and contraception use as well as a reduction in GBV. This trial is unique in a number of ways. First, the option of participation mode (SMS or VM) allows participants to choose the message medium that best links them to services. Second, this is the first RCT of a mobile-phone-based behavior change intervention using SMS/VMs to support linkage to SRH services in Cambodia. Lastly, we are working with a hidden, hard-to-reach, and dynamic population with which existing methods of outreach have not been fully successful.

**Trial registration:**

Clinical trials.gov, NCT03117842. Registered on 31 March 2017.

**Electronic supplementary material:**

The online version of this article (10.1186/s13063-018-2614-7) contains supplementary material, which is available to authorized users.

## Background

Mobile phones are increasingly being used to enhance the effectiveness of health programs by engaging individuals in discreet and ongoing ways. Mobile phone health interventions, called mHealth, can also improve the availability and quality of operational research data. mHealth interventions have been used successfully in developing countries to collect health data [1, 2], increase access to health knowledge [3–7], and increase medication/appointment adherence [8–11]. But, few mHealth interventions have been rigorously evaluated [6, 12]. There is strong evidence that mobile phone messages can be successfully used to support preventative healthcare [3, 5, 8, 12–15], such as maternal postpartum visit attendance and prevention of maternal-to-child transmission (PMTCT) of HIV in Kenya [16] and HIV prevention in India [17]. There is also evidence that self-report of risk behaviors through a mobile phone is a method of data collection that is as reliable and valid as in-person surveys [18]. Especially for mobile or hidden populations in developing countries, mobile phones may become our most important tool for gathering real-time health information and then linking people to health services [4, 5, 9, 17].

In Cambodia, several successful mobile-phone-based health programs have been introduced in the areas of post-abortion contraceptive use [19], pharmacovigilence [20], diabetes self-management [21], tuberculosis case detection/notification [22], and HIV education [23]. The current evidence base for mobile phones used for health assessment and encouragement of the uptake of services, especially for hard-to-reach populations that face stigma and discrimination in developing countries, is promising, but stronger impact evaluations are needed to understand the full benefits of such interventions.

One important hard-to-reach key population in Cambodia is the growing population of female entertainment workers (FEWs). FEWs are women working in entertainment venues such as karaoke bars, massage parlors, restaurants (as hostesses or singers), or beer gardens [24, 25]. FEWs frequently sell sex, directly or indirectly, to male patrons to supplement their income [24, 25]. By 2013, there were approximately 40,000 FEWs in Cambodia, 24,000 of whom resided in the capital city of Phnom Penh [26]. This group has been increasing since the 2008 passage and implementation of the “Law on Suppression of Human Trafficking and Sexual Exploitation,” which banned brothel-based sex work, more women have moved into indirect sex work based out of entertainment venues.

The influx in the number of FEWs also follows the increase in garment factory jobs that attract young women from poor rural families to migrate to urban areas to earn and remit income to their families in their home villages [24, 27]. Today, the garment factories in Cambodia employ over 700,000 young women [28]. Garment workers endure unsafe work environments [29] and claiming worker rights is fraught with dangers. As a result, more and more young migrant women become involved in alternative work opportunities in the entertainment industry. In addition, urban youth may also be at risk of engaging in sex work. A recent study from Cambodia found that 48% of at-risk female youth across the country reported having been paid for sex in the past year [30].

FEWs in Cambodia are considered at high risk for poor sexual and reproductive health (SRH) outcomes because of their involvement in direct or indirect sex work and limited access to SRH services [24, 30]. Recent studies indicate that HIV prevalence among women working in other entertainment establishments (9.8%) is lower than street-based sex workers (37.3%) and brothel-based sex workers (17.4%), but is still considered a national concern [31]. Recent data showed HIV incidence among FEWs was 9.2% in 2009 [32]. In addition, incidences of *C. trachomatis*, HPV, and *N. gonorrhea* among FEWs are 11.5%, 41.1%, and 7.8%, respectively [33, 34].

Recent surveys on HIV testing rates indicate that 81.7% of FEWs reported ever having had an HIV test, and 52.3% reported having had an HIV test in the past six months [35]. While there have been increases in reported condom use in commercial sex work over the past decade, there may be misreporting of condom use as well as condom failure. Consistent condom use is highly variable depending on the nature of relationship and several other factors [36, 37]. In 2014, our study of 667 FEWs showed that, in the past three months, 63.9% were able to find condoms when needed, and 38.2% used condoms at last sex with non-commercial partners [35]. Consistent condom use among FEWs and non-commercial partners, such as boyfriends or other romantic relationships, in the past three months is estimated to be 20.6–34.1% [33, 35, 38]. Moreover, recent studies have shown that 21.4% of FEWs reported having induced abortions since working in entertainment [31]. Sexually transmitted infections (STIs) are also common among FEWs [32–34].

In Cambodia, government and NGO providers currently offer free and widely available SRH services to FEWs. However, some FEWs experience barriers to using these services such as discrimination by providers. FEWs report a preference for private providers as a result [39]. KHANA, the largest national HIV organization in Cambodia, is currently offering specialized services for FEWs including free distribution of condoms, a “safe space” drop-in center, community-based finger-prick HIV and syphilis testing, accompanied referrals to enrolment of HIV-positive FEWs to pre-antiretroviral therapy (ART) and ART services, the integration of SRH services with HIV services, partner tracing, and PMTCT programming. Increasing utilization of these services by FEWs is a high priority.

The *Mobile Link* intervention is an operational mHealth research project that aims to engage FEWs and link them to the existing high-quality prevention, care, and treatment services in the country. This intervention will consist of theory-based text, i.e. short message service (SMS), or voice messages (VM), that aim to reduce risk behaviors and increase the linkage to and utilization of SRH and other related services among FEWs in Cambodia. In addition, this intervention may help to determine if SMS/VM messaging can be a reliable source of monitoring and evaluation data with this population. The initial operational research project will focus on FEWs as a proof of concept and, if effective, the *Mobile Link* intervention program will be tailored for other key populations.

### Cellphone use in Cambodia

Cambodia is the first country in the world where the number of mobile phone users has surpassed the number of fixed landlines [40]. The number of mobile subscribers in Cambodia reached 20 million at the end of 2013, surpassing the country’s population by about five million [41]. In a survey of 2597 youth aged 15–24 years drawn from all 24 provinces in Cambodia, 96% declared that they had access to a phone, with 68% reporting that they owned a personal mobile phone, and 72% had a mobile in their home [42]. The percentage of Cambodian people who own at least one smartphone was 39.5% in 2015, almost doubling the 2013 figure [43]. Controlling for location and gender, 51.3% of people aged 15–65 years in 2015 had at least one phone through which it was possible to send and receive messages in Khmer script (a 65% increase from last year) [44].

In a recent report from the United Nations Development Program (UNDP), of 2597 Cambodian people aged 15–24 years, 98% reported that they used their mobile phones mostly for making and receiving calls, 43% for listening to the radio or music, and 32% for sending and receiving messages, followed relatively closely by playing and downloading games (27%) and taking photos (22%) [42]. Of those youth who used SMS, the majority used English-language messages (81%) [42]. In the Asian-Pacific countries, the number of mobile social media users was set to reach one billion by the fourth quarter of 2015; and in Cambodia, increasing 108% from the previous year [45]. According to our recent observation study, FEWs have both smart and basic mobile phones, use them daily to text and engage in social media, and have expressed interest in receiving health information through their phones [46].

### Goal and objectives

The overall goal of the study is to deploy and rigorously evaluate the efficacy of the *Mobile Link*, an innovative intervention to engage young FEWs in Cambodia through frequent SMS/VMs that link them to existing high-quality HIV, SRH, and other related services. Specific objectives include: (1) developing the *Mobile Link*, a 12-month, two-arm randomized controlled trial (RCT), by conducting participant observation, focus group discussions (FGDs), in-depth interviews (IDIs), and revision workshops to produce a bank of theory-informed SMS/VMs as well as pilot testing the feasibility of collecting data from FEWs through SMS and VMs; (2) evaluating the efficacy of the *Mobile Link* at the individual and venue level in improving HIV and SRH behaviors and increasing the uptake of comprehensive HIV, SRH, and other related care services among sexually active FEWs; and (3) determining the cost-effectiveness of the *Mobile Link* for FEWs as compared to KHANA’s standard outreach, care, and treatment services.

## Methods

This manuscript is in accordance with the Standard Protocol Items: Recommended items to address in a clinical trial protocol and related documents (SPIRIT) guidelines (see Additional file 1 for SPIRIT checklist).

### Study design

**Fig. 1:**
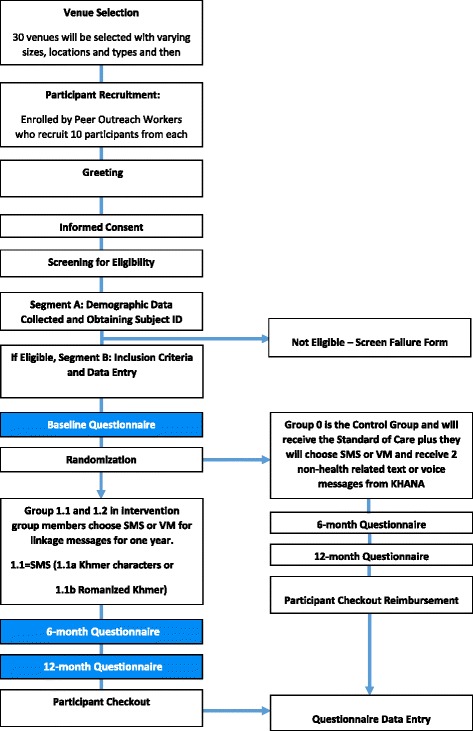
Study flow *diagram*

**Fig. 2:**
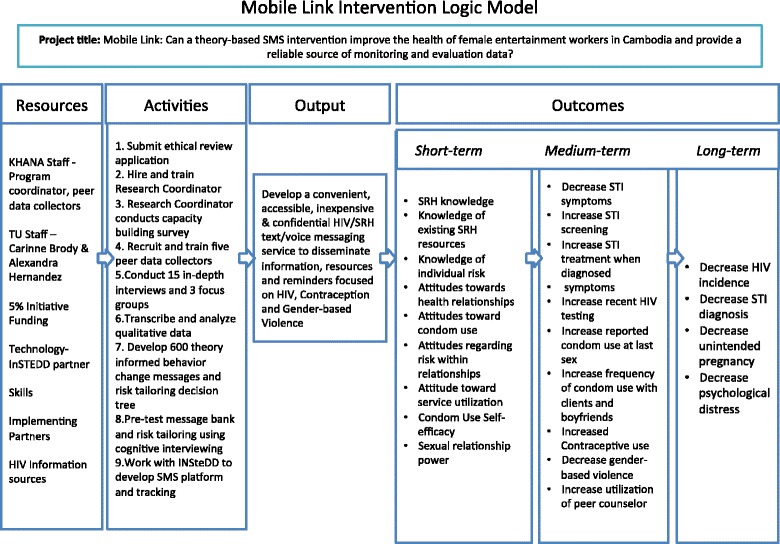
*Mobile Link* intervention logic model

The *Mobile Link* is a multisite, single-blind, 12-month RCT with two arms of 300 FEWs each (see Fig. 1). The trial will be conducted in the capital city of Phnom Penh and three provinces with high burden of HIV and a large proportion of FEWs including Battambang, Banteay Meanchey, and Siem Reap. The intervention would be developed following a review of the literature, formative research including surveys, interviews, and observations of FEWs, with inputs from public health professionals and technology partners in Cambodia. The *Mobile Link* conceptual framework (see Fig. 2) is based on the existing literature of the determinants of risk behaviors (i.e. unprotected sex) and utilization rates of prevention and treatment services (i.e. HIV and STI testing and treatment, contraceptive use).

### Participant selection criteria

All participants must: (1) work at an entertainment venue in the selected study sites; (2) be currently sexually active defined as having engaged in oral, vaginal, or anal sex in the past three months; (3) own a mobile phone; (4) know how to retrieve VM or retrieve and read SMS on a mobile phone (Khmer or Romanized Khmer); (5) self-identify as a FEW; (6) be willing to receive two SMS/VM per week for one year; (7) provide a written informed consent; and (8) agree to a follow-up visit after six months and 12 months. Figure 1 depicts participant flow diagram for the study.

### Theory of change

The logic model (Fig. 2) illustrates how the *Mobile Link* seeks to meet the goal of reducing the incidences of HIV, STIs, unintended pregnancies, and gender-based violence (GBV) among FEWs in Cambodia. It presents the flow of inputs and activities that are expected to lead to the stated short-, medium-, and long-term outcomes for the FEWs served. The first column of the logic model describes the inputs, including KHANA staff, faculty from Touro University California, funders, technology partnerships, skills, KHANA’s implementing partners, and HIV information sources that support program delivery. The activities include ethical approval, staff hiring, participant surveys, staff training, FGDs/IDIs, qualitative data analyses, message creation, message pre-testing, platform creation, questionnaire administration and data analyses (at baseline, midline, and endline), and message service delivery. Collectively, these make up the outputs. This output translates into short-, medium-, and long-term outcomes/impacts. Outputs offer knowledge. Ideally, this knowledge is of the services, existing resources, SRH, and risks within relationships. The positive attitudes toward SRH, SRH resources, risks within relationships and condom use, and negotiation, along with the knowledge will promote skill acquisition and positive behavior change including increased HIV and STI screening and treatment, consistent condom use with clients and/or boyfriends, contraception use, abortion, and experiences with GBV. In addition, lower experiences of STI symptoms, received STI diagnosis, self-reported HIV status should occur with higher utilization of peer counselors and utilization of any other services of KHANA's implementing partners. All of the knowledge, attitudes, skills, and behaviors will culminate in the impact/long-term outcomes of the program.

### Sampling and participant recruitment

**Fig. 3:**
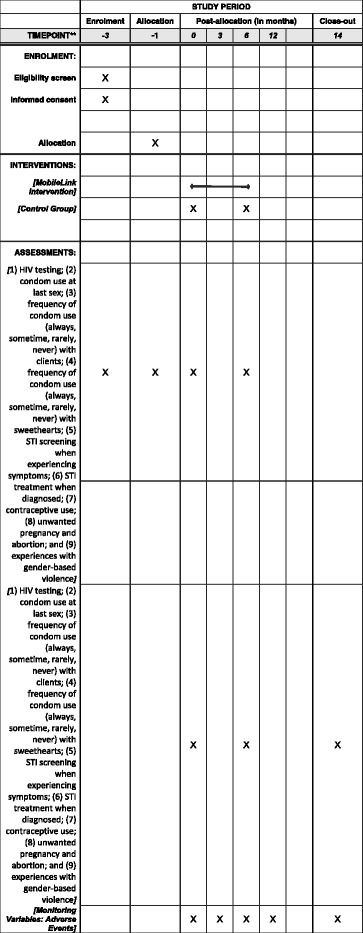
SPIRIT figure for *Mobile Link* protocol

We will use a stratified random sampling method to recruit survey participants. Venues will be selected from a list of all entertainment venues in the study sites based on a recent report on geographic information system (GIS) mapping of HIV key populations in Cambodia [47]. They are matched with 30 similar venues and then randomized for size and type of the venues. We will continue to sample from our list of all entertainment venues until we have a total of 600 FEWs recruited. All enrolled participants will complete an informed consent and a baseline questionnaire survey. Next, one venue from each venue pair will be randomly assigned to the intervention group: VM or SMS (1) and the other to the control group (0) with standard of care (no additional mobile phone-based SRH support) with a 1:1 venue allocation ratio. Both groups will be followed for one year, and a six-month and 12-month questionnaire will be administered by trained data collectors (see Fig. 3).

When approaching participants, recruiters will explain the details of the study and ask them for their informed consent to participate and enroll them in the study. If they agree, they will be asked questions regarding the inclusion/exclusion criteria. The community health workers will provide a list of all participants recruited, together with a unique trial identification number, to the program coordinator delivering the intervention. The community health workers will send only the ID number together with type of the entertainment venue of enrolled participants to the project statistician via email. Allocation will therefore be concealed from the researchers working on the trial. The enrollment procedures will also require them to give mobile numbers for all of their SIM cards, as many people in Cambodia have multiple SIM cards. We will also ask them which phone number is their most used one.

### Baseline data collection

All data collectors and field supervisors will attend a training session for two days covering necessary skills including interview techniques, confidentiality, and privacy assurance. The study protocol and tools will also be reviewed during the training sessions in order for the team members to be thoroughly familiar with it. We will also include quality control skills such as rechecking and reviewing the questionnaires after administration as well as resolving issues that might arise during the fieldwork. Team leaders will be encouraged to perform regular review sessions with interviewers during the survey period to review progress and communicate any issues occurring during the data collection.

Given the high rates of illiteracy in this community, the data collectors will verbally explain the study by reading the participant information provided as part of the informed consent process before starting FGDs and interviews. If a FEW wishes to participate, she would express her verbal consent (see Additional files 2 and 3). In addition, a witness that is not associated with the study will be present and will sign the informed consent form.

Data collectors, who will be blinded to the trial arm assignment, will collect baseline data from participants recruited, as well as survey them at six and 12 months. All participants will receive SMS/VM appointment reminders for the surveys and peer data collectors will meet participants in a convenient and private location to conduct the surveys. If a participant misses a scheduled study visit, data collectors will send SMS/VMs and make voice calls to re-schedule the missed visits. If unsuccessful in contacting the participant through those modes, they will arrive in person at the workplace in an attempt to follow-up with the participant.

### Intervention

#### Phase I: Iterative, participatory intervention development

In phase 1, a total of 27 FGDs were conducted across all four implementation sites and among all four entertainment venue types, including street-based and on-call sex workers (for FGD guides see Additional file 4). IDIs were held with program staff as well as with FEWs living with HIV. The FGD guides were semi-structured and explored a range of topics related to HIV, SRH, GBV, and substance use. Conversation and storytelling were encouraged. Recognizing potential variable experiences and needs across entertainment venue types and geographic areas, FGDs were conducted across venue types among all four sites to achieve saturation.

Following initial data analyses, messages have been created with a local NGO, Women’s Media Center, and presented to a selected group of FGD participants and outreach workers to revise and refine messaging. The findings from the FGDs and revision workshops highlighted that for many FEWs, health priorities such as adverse effects of high alcohol use (gastrointestinal issues) and gynecologic issues (STIs and vaginal infection/irritation) outweigh concerns around HIV and family planning. In fact, during the revision workshops, HIV was most often cited as an area that women did feel they had a good knowledge base and linkage to testing and treatment. Participants also reinforced through discussions a number of misconceptions related to contraception – particularly oral contraceptives and intrauterine devices (IUDs) – as well as HIV and STI transmission. Many of those misconceptions will be directly addressed through the *Mobile Link* messaging. Lastly, many participants spoke to the importance of outreach workers and the linkages that Smart Girl provides, further confirming the emphasis on *Link* within the *Mobile Link* program.

Based on the formative research, messages tagged within the following topic areas (acknowledging some crossover) will be sent to intervention participants: gynecologic health; condom use; HIV; STIs; contraception; substance use; violence; infertility; cancer; and pregnancy. Further and more detailed findings from the formative phase are in the process of being synthesized for dissemination. Using the message bank, the SMS/VM platform and tracking will be developed and optimized for program use in collaboration with local programmers at the InSTEDD iLab in Phnom Penh.

#### Phase II: Intervention

The *Mobile Link* intervention comprises a series of automated SMS/VMs to participants’ mobile phones over the 12-month period. Participants will receive the first message within the first day of the intervention. The messages will be designed to enhance and increase utilization of existing HIV and SRH services by reminding clients about safe sex methods available to them and providing a conduit for additional support. Upon enrollment into the study, participants in all groups will receive a face-to-face baseline behavioral survey conducted by a data collection team who remained masked to intervention allocation at enrollment.

The baseline questionnaire (see Additional file 5) was designed by the study team using sections from standard questionnaires currently in use at KHANA for assessing similar measures in populations of high-risk women in Cambodia. The questionnaire was created in English, translated to Khmer, and back-translated to English. The questionnaire will be pilot tested with 15 FEWs similar in eligibility to future study participants using cognitive interviewing techniques [48]. Next, participants will be randomized into intervention and control groups: Group 0 is the control group and Group 1.1 and 1.2 are the intervention group (1.1 = SMS [1.1a Khmer characters or 1.1b Romanized Khmer] or 1.2 = VM in Khmer).

Those in the control group will get one “check-in” SMS or VM between baseline and midline and another between midline and endline. Participants will be asked to confirm receipt of this message. These messages will contain no health information and will be used as a way to stay in touch with participants and to compare “receipt confirmation” rates to the intervention group. All participants will be asked to choose whether they would prefer to receive SMS or VM and their reason for their choice. If they choose SMS, they will be asked if they prefer Khmer characters or Romanized Khmer. Recent studies on choosing the method for receiving interventions during an RCT have demonstrated that it may increase participation and retention [49].

A message schema was developed in which each participant will receive a message from a different topic group each week for 10 weeks. The messages will tend to follow a flow of rights-based health information and then linkage (though there is some variance depending on topic). However, after each message, participants will also have the option to be linked to an outreach worker. After the tenth week, the topic areas will cycle through again, but the messages within the topic areas will be unique. After weeks 30, 40, and 50 (+ 2), messages will be repeated to reinforce particular concepts and ideas. Participants who indicate they would like to talk to a counselor will receive a call from a *Mobile Link* staff. The counselor will provide individualized information via telephone or face-to-face on that particular topic and, if needed, can escort the participant to receive the required services.

Participants will also receive a weekly 5–7-question survey that asks about more common and/or time-sensitive issues such as STI symptoms, vaginal infection/irritation, support for GBV, or psychological issues. Once a month, participants will be asked if they are using contraception and/or want to be linked to family planning counseling. Participants who endorse any of the survey questions will be asked if they want to be linked to an outreach worker. Therefore, it is important to note that, while the topics in the messages will change each week—as well as the topical prompts for linkages to outreach workers—the weekly monitoring survey provides another consistent and constant platform for mobile linkage.

Trained data collectors and community health workers in the selected sites will deliver the *Mobile Link* intervention. SMS/VM are scheduled and sent using the open-source software program developed by InSTEDD (instedd.org). InSTEDD and the Project Coordinator will retain data on the SMS/VM messages sent, responses to messages, and outcomes of follow-up phone calls.

#### Phase III: Cost-effectiveness analyses

All relevant costs related to developing and implementing the intervention, minus the cost caused by the study, will be collected. This will include the cost of developing the SMS/VM platform as well as all the fixed and variable costs of running the intervention for one year. We will look at the cost of engaging and linking participants but will not include the cost of the health services that participants use as a result of the program. In that way, we are looking at the cost-effectiveness of adding the SMS/VM component to SRH services. We will use five measures of effect that will be self-reported by participants during baseline and follow-up surveys: uptake of first HIV test (0 to1); uptake of regular HIV test (at every six months for those engaging in unprotected sex with sweethearts or engaged in sex work); uptake of first visit to a SRH care provider for any testing services or counselling; uptake of any visits to a SRH care provider for any testing, services, or counselling; and uptake of condom use at last sex.

An average cost-effectiveness ratio (CER) will be calculated by dividing the total cost of each arm by the change in outcomes of each arm. In addition, the incremental CER (ICER) will be calculated, which will measure the cost per additional HIV test/visit/protected sex under the *Mobile Link* as compared to no SMS/VM intervention.

#### Control group

Participants in the control group will receive the current existing standard of care, but not the health-related SMS/VMs or associated follow-up phone calls. Standard of care consists of access to existing HIV and SRH services which includes: face-to-face counseling; free HIV and STI testing and condoms; and clinic phone numbers and hotline phone numbers with a toll-free help line for clients staffed by trained counselors. Control group members will receive “check-in” reminder calls before questionnaire appointments (midline and endpoint).

### Primary outcomes

All outcomes will be self-reported and collected through surveys. The primary outcome measures will include: (1) HIV testing; (2) condom use at last sex; (3) frequency of condom use (always, sometime, rarely, never) with clients; (4) frequency of condom use (always, sometime, rarely, never) with boyfriends; (5) STI screening when experiencing symptoms; (6) STI treatment when diagnosed; (7) contraceptive use; (8) unwanted pregnancy and unsafe abortion; and (9) experiences with GBV.

### Secondary outcomes

The secondary outcome measures will include: (1) knowledge of SRH; (2) knowledge and attitudes toward existing SRH resources; (3) knowledge and attitudes toward abortion laws and services; (4) knowledge and attitudes regarding risk within relationships; (5) attitudes toward condom use and condom negotiation; (6) experiences of STI symptoms; (7) received STI diagnosis; (8) self-reported HIV status; (9) utilization of peer counselor; and (10) utilization of any other SRH services.

### Data collection

Data collection tools will include baseline and follow-up questionnaires administered by a data collection team. The baseline questionnaire will contain questions to collect information on contact details; demographics; pathways to entertainment work; information about their current workplace; SRH knowledge, attitudes, and behaviors; sexual relationships; condom use self-efficacy; and experiences with GBV. All questions will be asked in both written and oral forms because literacy rates are not 100% in this community. In addition, all data collected via weekly monitoring surveys will be analyzed on a routine basis.

### Data management

Data coding, quality control, and data entry will be done following established procedures at KHANA. Pre-coded questionnaire will be used to minimize data coding errors. All data forms and questionnaires will be checked for errors by a second data collector; errors and necessary corrections will be made before data entry. A database will be developed for data entry in MS Access, which will include built-in range and within- and between-variable consistency checks. The program will also run error-checking applications and produce reports of inconsistencies to be checked daily.

### Analysis plan

#### Analyses of participant characteristics

Participant characteristics and survey responses will be analyzed using descriptive statistics including Chi-square test of homogeneity and paired Student’s *t*-tests to compare characteristics between the participants to ensure balance among the study arms.

#### Analyses of primary and secondary outcomes

We will report the trial according to the Consolidated Standards of Reporting Trials (CONSORT) standards for reporting RCTs. This is a behavioral intervention unlikely to produce adverse effects, so analysis will be undertaken once the six-month follow-up has been completed.

The effect of the intervention will be measured through repeated measures generalized linear models estimated through generalized estimating equations (GEE) linear/logistic regression (as appropriate) accounting for potential correlation from taking repeated measures on participants. Separate models will be conducted for each of the outcomes. Predictors in the models will include intervention group assignment, time, and group by time interaction terms. The intervention will be considered effective if the group by time interaction has a significant *p* value (*p* ≥ 0.05).

Intention-to-treat (ITT) principles will be used for primary outcome analyses; therefore, all participants will be analyzed according to the arm to which they were randomized. During ITT analyses, participants lost to follow-up, resulting in missing outcome data at six months, will be considered non-users.

For the primary and secondary outcomes, we will use GEE with covariates to estimate effect. We will calculate the sensitivity and specificity of self-reported contraception use as compared to objective measurement and comment on any limitations of the respective methods of data collection. We will undertake Kaplan–Meier survival analysis to compare contraceptive discontinuation rates. Analyses will be conducted using STATA.

#### Sensitivity and per-protocol analyses

We will conduct an additional sensitivity analysis including only participants who completed the six-month follow-up. Per-protocol analysis will be undertaken to assess the impact of the intervention among those who actively participated in the intervention. Participants who respond to > 50% of the six VM/SMS over the intervention period will be considered highly protocol-adherent. Participants who respond to 0.5–50% will be considered less protocol-adherent. Those who never responded to a voice message will be considered as never-responding and not included in the sensitivity analysis.

#### Sub-group analyses

We will undertake exploratory sub-group analyses to assess evidence for whether the effect of the intervention varies according to age, entertainment venue, level of education, and socioeconomic status. If statistically significant, overall heterogeneity is identified, then relative risks and 99% confidence intervals will be estimated.

#### Additional analyses

We will provide a descriptive analysis of information on study-related mobile phone use among participants. The aim will be to include number of voice message and phone interactions, response to voice messages, response to weekly surveys, time spent on phone calls, and linkages to outreach workers to facilitate description of process indicators and any issues. Additionally, at the end of the trial, the costs of the intervention (training, human resources, phone costs, and so on) will be estimated.

#### Qualitative interviews

We will conduct around 15 to 20 qualitative interviews (or until data saturation has been reached) with participants who received the intervention. Participants for interviews will be selected purposively to include those who did or did not appear to respond to the intervention. The interviews will explore participants’ experience of the intervention, aiming to identify active components of the intervention, and seek recommendations for improvements. Interviews will be recorded and transcribed and a simple thematic analysis will be undertaken. We will use the findings to inform any adjustments to the intervention after the trial. We will also conduct process interviews with community health workers and outreach workers to better understand the utility and effectiveness of the weekly monitoring survey in their work and explore feasibility of scaling.

### Ethical consideration

The trial will be conducted in accordance with the principles of Good Clinical Practice. The study protocol has been approved by the National Ethics Committee for Health Research (NECHR, No. 142NECHR) of Ministry of Health in Cambodia and Touro College Institutional Review Board (No. PH-0117). The *Mobile Link* trial has been registered with ClinicalTrials.gov (No. NCT03117842).

All participants will provide verbal informed consent before enrolling in the trial or commencing the follow-up interviews. All client records and data will be stored securely. No names of participants (or others mentioned) or locations will be used in the analyses or report writing. Confidentiality will be maintained by assigning coded identifiers to participant names (with a master list stored separately). Participants will be able to withdraw from the study at any point. When withdrawing from the study, the participant would let the research team know that she wishes to withdraw. The participant may provide the research team with the reason for leaving the study, but this is not requirement.

Any participants with an adverse outcome arising from other people listening to VM (for example, an argument or violence) will be linked into appropriate existing services at local organizations. Each survey will ask about experiences with social harms as a result of the study. Social harms experienced by participants and reported to research staff during data collection will be documented and monitored by investigators. Participants will be reimbursed to compensate for expenses related to face-to-face clinic follow-up and for participation in the surveys.

#### Application, dissemination, and transposition of results

This study is a proof of concept. If found to be both effective and cost-effective, replication and rapid scale-up of the intervention can be achieved. The nature of the intervention, SMS/VM interactions, can be easily modified using the existing digital platform developed for this trial to accommodate more users from the same population after performing outreach and enrolment. In order to serve users from a different population, we would need to conduct qualitative work to develop new tailored high-quality theory-informed messages and conduct the initial outreach and enrolment activities. The results of this study will be shared at local, regional, and international levels. We will collaborate with national institutions, such as the National Center for HIV/AIDS, Dermatology and STD (NCHADS), the National AIDS Authority, and the National Institute of Public Health (NIPH) in Cambodia, who will be aware of and involved in this project, to disseminate the information about this study locally. We would plan to host a regional meeting with stakeholders from neighboring countries, such as Laos, Thailand, and Vietnam, who have similar at-risk populations in order to share our results. We plan to share results at international conferences and through peer-reviewed journal article publications.

### Potential risks and benefits

#### Human participants

This study will involve human participants and their protection is in accordance with the NECHR and the Touro College Institutional Review Board. NECHR reviews research proposals involving human participants with a view to safeguard the dignity, rights, safety, and wellbeing of all actual or potential research participants. They state that the goals of research, however important, should never be permitted to override the health and wellbeing of the research individuals. The Touro College Institutional Review Board aims to protect the rights and welfare of human participants recruited to participate in research activities conducted under the auspices of the College. The Board is registered with the federal Office of Human Research Protections (OHRP) and received approval of its Federal Wide Assurance (FWA) on 30 April 2008.

All key personnel involved in this study will be required to complete the online National Institute of Health (NIH) course on the protection of human research participants and to show proof of course completion which will be kept on file. No research or recruitment of individuals will begin without approval of the two ethical review boards mentioned above.

### Risks to human participants

#### Human participant involvement, characteristics, and design

The focus of this study is to increase the rates of HIV testing and condom use in FEWs because they have been identified as a group that is at-risk for HIV infection. Female young adults who are classified as a vulnerable population comprise most of the study population. Because this study is testing an intervention tailored specifically to females, men will not be eligible to be participants. All recruitment activities and study procedures involving study participants will follow national guidelines. We anticipate involving a minimum of 600 FEWs in the study, and our sampling strategy will ensure that the group of participants is representative of the total population of young FEWs in Cambodia. Assigning study arms will be done at random, meaning that all participants have an equal chance of being assigned into the two active arms or the control arm. They will not be masked to the intervention since the intervention involves receiving SMS/VMs on their mobile phones.

#### Sources of materials

As stated in the study protocol, research materials will include interview digital audio files and transcripts, field observation notes, survey responses, and SMS/VM response data. All data obtained through FGDs, in-person questionnaires, or SMS/VM data collection will be de-identified and stored in password-protected electronic files as well as in a hard copy format in locked file cabinets at the KHANA’s office in Cambodia and at Touro University California and will only be accessible by the Principal Investigators and Co-Investigators.

#### Potential risks

The risks to participation in this study include psychological distress as a result of answering questions about sensitive subjects and a breach of confidentiality as a result of receiving the SMS/VMs about sensitive topics on their mobile phones. This may put participants at risk of negatively affecting their occupational, personal, or romantic relationships if someone else sees the messages. For those participating in FGDs, there may be an infringement on privacy if participants feel pressured or get caught up in the moment and disclose personal information to people they may know or see in their workplaces or communities. To address these risks, all study participants will be informed about the voluntary nature of their participation and their rights to end the participation at any point during the study without any consequences. They will have access to free youth-friendly counseling services and will also be given information about crisis centers and other relevant referrals, and they will be offered escorted referrals.

### Adequacy of protection against risks

#### Recruitment and informed consent

Participants will be recruited by community health workers. Before participation in any study activities, a verbal consent will be sought from the study participants. Data collectors will sign the consent form to confirm that the study participant has been briefed about the study, assured of their confidentiality, and have given their informed consent to participate in the study. Study participants will also be informed of the option for escorted referrals to appropriate existing government and non-government services in the province if they so wished. Because this research topic is highly stigmatizing, verbal consent is the most appropriate form of consent so that no other identifying information is collected. For those study participants who cannot read, we will identify individuals who work in the outreach programs but are not part of the research team to act as witnesses and to sign the consent form.

#### Risk for vulnerable groups

We will be enrolling vulnerable populations and participants who may be pregnant or become pregnant during the study. Pregnancy does not exclude participants from the study but is not the target group of this research. All activities with these groups meet the procedural guidelines of our institutional review board. There are no known risks to the health of pregnant women or their fetuses or neonates of participating in this study. In addition, there may be benefits to them because FEW-specific SRH services will be made available to them throughout the study period, and trained community health workers will offer them free escorted referrals to SRH services.

#### Confidentiality and data monitoring

The records of this study will be kept confidential. The research team will not include the name or personal identifiers of any participants involved in the research in our reports. The interview recordings, questionnaires, and recordings obtained from the participants will be safely locked away in a digital recorder with a passcode. Electronic transcripts will be stored on a password-protected laptop. Informed consent forms and demographic questionnaires with identifiable information will be stored in a locked bag during transportation to and from the study sites. These forms will be kept in a separate locked file away from digital recordings and transcripts. The research coordinator is the only person who will have access to these records.

Data monitoring and interim analyses at the six-month and one-year follow-up will be conducted by the lead investigators of the research team. At that time, the team will determine if the trial should be continued as planned, stopped early for hazard (including social harms), or stopped early because efficacy is overwhelmingly established or the trial is futile.

### Costs of participation

The *Mobile Link* will incur the cost of outgoing communication from providers to participants; participants incur any costs calling or messaging into the program and services. However, participants in the intervention arm will receive $5 as a compensation after completion of the intervention.

### Compensation and incentives

Participants in the FGDs will be offered $5 for participation in each FGD. Participants in the questionnaire surveys at baseline, midline, and endline will be offered $5 for every survey they complete.

### Assumptions, risks, and preconditions

Mobile phone projects in developing countries are not without limitations; technical and operational challenges must be addressed for our programs to work. In terms of technical limitations, mobile users often own multiple SIM cards in order to get cheaper in-network rates and better reception from the five or six very competitive networks in Cambodia who also offer deals that entice users to use their SIM for a limited period of time. In addition, many people share phones with family members or even neighbors and therefore may not receive health-related messages or phone calls or may not sign up for programs out of concern that others would receive their private information, although this is more common in rural areas. Level of literacy is another limiting factor when trying to reach broad segments of the population in developing country contexts with SMS/VM, and many projects have turned to VM to circumvent this issue.

In Cambodia, there is the additional concern that phones lack the ability to send SMS in Khmer script, although the younger generation of tech-savvy Cambodians is more familiar with a Romanized Khmer language used for texting and social media. Other mHealth interventions that have shown promise have faced operational challenges that limit the ability to take them to scale. Common errors include using interventions that are at a cost or complexity level that are too high for scale such as relying on smartphones. Other issues include not designing programs for their end users that can, for example, give overburdened community health workers or peer counselors more work instead of less.

We have addressed these concerns in the following ways. First, we will use a technology (SMS/VM) that can be used by both simple and smart phone users. Second, we have collected preliminary data from our population of interest, which indicated that sending and receiving SMS/VM in Khmer or Romanized Khmer is acceptable to FEWs. Lastly, we have collected preliminary data from our population of interest, which indicate that FEWs are able to receive personal and private messages from community health workers. We are planning to collect phone numbers associated with all SIM cards in use by each participant so as to increase the chances that they will receive each message.

## Discussion

Finding better strategies for linking hard-to-reach populations who are at risk for HIV to quality health services is a global health priority. The World Health Organization (WHO) has stated that a worldwide failure to provide adequate HIV services for key populations such as men who have sex with men, people in prison, people who inject drugs, sex workers, and transgender people may be a barrier to achieving progress toward reversing the HIV epidemic [50]. Previous studies have highlighted the unique challenges to linking key populations to HIV services in Asia [32, 33, 37].

In Cambodia, the 2008 “brothel ban” and consequent surge in indirect sex work has created additional barriers to connecting vulnerable and high-risk FEWs with health services. The *Mobile Link* trial aims to link FEWs to services and increase risk-reducing behaviors using a technology women use daily to connect with family and friends [42, 44, 51]. Weekly messages tailored to their specific needs may improve their knowledge and attitudes toward connecting with services and promote positive behavior change.

We anticipate several limitations associated with recruitment, retention, and measurement in this study. Our study population is a hidden, hard-to-reach group that is mobile, at least across entertainment venues. As such, we anticipate challenges in recruiting and following-up with participants over the 12-month study period. We will make efforts to address these limitations by providing incentives for participation, making frequent contact through mobile phones, collecting phone numbers from all currently owned SIM cards at baseline and midline, and asking for the contact information for their next nearest contact.

Another concern is the broad range of literacy rates within our study population which may affect the quality of participation and length of retention. In our feasibility studies, we tested small groups of FEWs for reading and writing literacy [35]. Most women received the highest literacy rating but several received the lowest rating. In addition, KHANA staff have reported that they have encountered problems with reading comprehension within this population. For this reason, the VM option within the intervention arm of the study was added. Recent evidence suggests that offering a choice of participation mode within an RCT can improve both recruitment and retention [50].

This trial is being conducting in an extremely dynamic environment. Rapid changes in sex work practices, the mobile phone industry, and the way youth and young adults use technology may result in external validity issues that may prove to limit the representativeness of the data. In order to address this issue, our study period will be only 12 months and include baseline, midline, and endline data collection points that can alert us to any large changes within that time.

Finally, as HIV testing is a primary outcome and HIV status is a secondary outcome, it was decided the secondary outcome of HIV status would be self-reported as incorporating into the trial an HIV test to conclusively determine status may bias the primary outcome HIV testing data. Understanding the factors surrounding the decision to get tested for HIV is central to the core of this research and therefore HIV status data will be limited to self-reported information.

The findings of this novel study will strengthen our understanding of how mHealth interventions may be used to link key populations to health services in Cambodia and in other developing countries.

## Trial status

In development stage, recruitment not started.

## Additional files

Additional file 1: SPIRIT checklist. (DOC 122 kb)

Additional file 2: Informed consent form for focus group discussions and in-depth interviews. (DOCX 20 kb)

Additional file 3: Informed consent for questionnaire survey. (DOCX 21 kb)

Additional file 4: Focus group discussion and in-depth interview guides. (DOCX 18 kb)

Additional file 5: Questionnaire for baseline survey. (DOCX 75 kb)

## Abbreviations

AIDS: Acquired immune deficiency syndrome
ART: Antiretroviral therapy
CER: Cost-effectiveness ratio
FEW: Female entertainment workers
FWA: Federal Wide Assurance
GEE: Generalized estimating equation
GIS: Geographic information system
HIV: Human immunodeficiency virus infection
IP: Implementing partner
IRB: Institutional review board
ITT: Intention to treat
NCHADS: National Center for HIV/AIDS, Dermatology and STD
NECHR: National Ethics Committee for Health Research
NIPH: National Institute of Public Health
OHRP: Office of Human Research Protections
PMTCT: Prevention of mother-to-child transmission
RCT: Randomized controlled trial
SMS: Short message service
SRH: Sexual and reproductive health
STD: Sexually transmitted diseases
STIs: Sexually transmitted infections
UNDP: United Nations Development Programme
VM: Voice message
WHO: World Health Organization

## References

[1] Gisore P, Shipala E, Otieno K, Rono B, Marete I, Tenge C (2012). Community based weighing of newborns and use of mobile phones by village elders in rural settings in Kenya: a decentralized approach to health care provision. BMC Pregnancy Childbirth.

[2] Curran K, Mugo NR, Kurth A, Ngure K, Heffron R, Donnell D (2013). Daily short message service surveys to measure sexual behavior and pre-exposure prophylaxis use among Kenyan men and women. AIDS Behav.

[3] Hossain M, Mani KK, Sidik SM, Hayati KS, Rahman AK (2015). Randomized controlled trial on drowning prevention for parents with children aged below five years in Bangladesh: a study protocol. BMC Public Health.

[4] Crawford J, Larsen-Cooper E, Jezman Z, Cunningham SC, Bancroft E (2014). SMS versus voice messaging to deliver MNCH communication in rural Malawi: assessment of delivery success and user experience. Glob Health Sci Pract.

[5] Dammert AC, Galdo JC, Galdo V (2014). Preventing dengue through mobile phones: Evidence from a field experiment in Peru. J Health Econ.

[6] Free C, Phillips G, Galli L, Watson L, Felix L, Edwards P (2013). The effectiveness of mobile-health technology-based health behaviour change or disease management interventions for health care consumers: a systematic review. PLoS Med.

[7] Déglise C, Suggs LS, Odermatt P (2012). Short message service (SMS) applications for disease prevention in developing countries. J Med Internet Res.

[8] Mushamiri I, Luo C, Iiams-Hauser C, Ben AY (2015). Evaluation of the impact of a mobile health system on adherence to antenatal and postnatal care and prevention of mother-to-child transmission of HIV programs in Kenya. BMC Public Health.

[9] Raifman JR, Lanthorn HE, Rokicki S, Fink G (2014). The impact of text message reminders on adherence to antimalarial treatment in northern Ghana: a randomized trial. PLoS One.

[10] Sabin LL, Bachman DeSilva M, Gill CJ, Zhong L, Vian T, Xie W (2015). Improving adherence to antiretroviral therapy with triggered real-time text message reminders: The China Adherence Through Technology Study. J Acquir Immune Defic Syndr.

[11] Islam SM, Lechner A, Ferrari U, Froeschl G, Alam DS, Holle R (2014). Mobile phone intervention for increasing adherence to treatment for type 2 diabetes in an urban area of Bangladesh: protocol for a randomized controlled trial. BMC Health Serv Res.

[12] Vodopivec-Jamsek V, de Jongh T, Gurol-Urganci I, Atun R, Car J (2012). Mobile phone messaging for preventive health care. Cochrane Database Syst Rev.

[13] Esbensen BA, Thomsen T, Hetland ML, Beyer N, Midtgaard J, Løppenthin K (2015). The efficacy of motivational counseling and SMS-reminders on daily sitting time in patients with rheumatoid arthritis: protocol for a randomized controlled trial. Trials.

[14] Youl PH, Soyer HP, Baade PD, Marshall AL, Finch L, Janda M (2015). Can skin cancer prevention and early detection be improved via mobile phone text messaging? A randomised, attention control trial. Prev Med.

[15] Smith C, Vannak U, Sokhey L, Ngo TD, Gold J, Free C (2016). Mobile Technology for Improved Family Planning (MOTIF): the development of a mobile phone-based (mHealth) intervention to support post-abortion family planning (PAFP) in Cambodia. Reprod Health.

[16] Odeny TA, Bukusi EA, Cohen CR, Yuhas K, Camlin CS, McClelland RS (2014). Texting improves testing: a randomized trial of two-way SMS to increase postpartum prevention of mother-to-child transmission retention and infant HIV testing. AIDS.

[17] Swendeman D (2013). Are mobile phones the key to HIV prevention for mobile populations in India?. Indian J Med Res.

[18] Swendeman D, Comulada WS, Ramanathan N, Lazar M, Estrin D (2015). Reliability and validity of daily self-monitoring by smartphone application for health-related quality-of-life, antiretroviral adherence, substance use, and sexual behaviors among people living with HIV. AIDS Behav.

[19] Smith C, Vannak U, Sokhey L, Ngo TD, Gold J, Khut K (2013). MObile Technology for Improved Family Planning Services (MOTIF): study protocol for a randomized controlled trial. Trials.

[20] Baron S, Goutard F, Nguon K, Tarantola A (2013). Use of a text message-based pharmacovigilance tool in Cambodia: pilot study. J Med Internet Res.

[21] van Olmen J, Ku GM, van Pelt M, Kalobu JC, Hen H, Darras C (2013). The effectiveness of text messages support for diabetes self-management: protocol of the TEXT4DSM study in the democratic Republic of Congo, Cambodia and the Philippines. BMC Public Health.

[22] Lorent N, Choun K, Thai S, Kim T, Huy S, Pe R (2014). Community-based active tuberculosis case finding in poor urban settlements of Phnom Penh, Cambodia: a feasible and effective strategy. PLoS One.

[23] Wright G, Odom S (2015). Mobile phone program aims to reach HIV-vulnerable groups.

[24] Union Aid Abroad–Australian People for Health, Education and Development Abroad (APHEDA) (2011). Cambodia—addressing HIV vulnerabilities of indirect sex workers during the financial crisis: situation analysis, strategies and entry points for HIV/AIDS workplace education.

[25] Brody C, Chhoun P, Tuot S, Pal K, Chhim K, Yi S (2016). HIV risk and psychological distress among female entertainment workers in Cambodia: a cross-sectional study. BMC Public Health.

[26] National Center for HIV/AIDS, Dermatology and STD (NCHADS) (2013). Annual report 2012.

[27] Webber G, Edwards N, Grahamet ID, Amaratungad C, Keanee V, Ros S (2010). Life in the big city: the multiple vulnerabilities of migrant Cambodian garment factory workers to HIV. Womens Stud Int Forum.

[28] Prak CT, Pearce T (2015). Cambodia and big brands fail to tackle garment worker abuse: researchers.

[29] Loomis E. Out of Sight: The Long and Disturbing Story of Corporations Outsourcing Catastrophe. New York: The New Press; 2015.

[30] Brody C, Tuot S, Chhea C, Saphonn V, Yi S (2016). Factors associated with sex work among at-risk female youth in Cambodia: a cross-sectional study. AIDS Care.

[31] Yi S, Tuot S, Chhoun P, Pal K, Tith K, Brody C (2015). Factors associated with induced abortion among female entertainment workers: a cross-sectional study in Cambodia. BMJ Open.

[32] Page K, Stein E, Sansothy N, Evans J, Couture MC, Sichan K (2013). Sex work and HIV in Cambodia: trajectories of risk and disease in two cohorts of high-risk young women in Phnom Penh, Cambodia. BMJ Open.

[33] Couture MC, Sansothy N, Sapphon V, Phal S, Sichan K, Stein E (2011). Young women engaged in sex work in Phnom Penh, Cambodia, have high incidence of HIV and sexually transmitted infections, and amphetamine-type stimulant use: new challenges to HIV prevention and risk. Sex Transm Dis.

[34] Couture MC, Page K, Stein ES, Sansothy N, Sichan K, Kaldor J (2012). Cervical human papillomavirus infection among young women engaged in sex work in Phnom Penh, Cambodia: prevalence, genotypes, risk factors and association with HIV infection. BMC Infect Dis.

[35] Brody C, Tuot S, Chhoun P, Pal K, Chhim K, Yi S. Recent HIV testing and associated risk factors among female entertainment workers in Cambodia. PLoS One. 2017; (In Press) 10.1371/journal.pone.0198095 PMC6028079 29965968

[36] Yi S, Tuot S, Chhoun P, Brody C, Tith K, Oum S (2015). The impact of a community-based HIV and sexual reproductive health program on sexual and healthcare-seeking behaviors of female entertainment workers in Cambodia. BMC Infect Dis.

[37] Maher L, Mooney-Somers J, Phlong P, Couture MC, Kien SP, Stein E (2013). Condom negotiation across different relationship types by young women engaged in sex work in Phnom Penh, Cambodia. Glob Public Health.

[38] Bui TC, Markham CM, Tran LT, Beasley RP, Ross MW (2013). Condom negotiation and use among female sex workers in Phnom Penh, Cambodia. AIDS Behav.

[39] Ministry of Education, Youth, and Sport (MEYS) (2010). Examining life experiences and HIV risks of young entertainment workers in four Cambodian cities.

[40] International Development Research Center (IDRC). Digital Review of Asia Pacific 2007/2008. Ottawa: IDRC; 2008.

[41] Ben S (2014). Mobile users top 20 million, internet usage still rising.

[42] BBC Media Action (2014). Youth in Cambodia: Media habits and information sources.

[43] Rollet C (2015). Smartphone ownership exploded since ‘13: study.

[44] Phong K, Solá J (2014). Mobile phones in Cambodia 2014.

[45] Baliga A (2015). Cambodians flock to the net.

[46] Brody C, Tatomir B, Sovannary T, Pal K, Mengsrun S, Dionosio J (2017). Mobile phone use among female entertainment workers in Cambodia: an observation study. Mhealth.

[47] National Center for HIV/AIDS, Dermatology and STD (NCHADS) (2014). Report on the first comprehensive GIS mapping of key populations at risk of HIV Infection in Cambodia.

[48] Willis GB (1999). Cognitive Interviewing: A “How To” Guide.

[49] Heijmans N, van Lieshout J, Wensing M (2015). Improving participation rates by providing choice of participation mode: two randomized controlled trials. BMC Med Res Methodol.

[50] World Health Organization (WHO). WHO: People most at risk of HIV are not getting the health services they need. Geneva: WHO; 2014.

[51] Brody C, Dhaliwal S, Tuot S, Johnson M, Pal K, Yi S (2016). Are text messages a feasible and acceptable way to reach female entertainment workers in Cambodia with health messages? A cross-sectional phone survey. JMIR Mhealth Uhealth.

