# Correction to: Mobile Link – a theory-based messaging intervention for improving sexual and reproductive health of female entertainment workers in Cambodia: study protocol of a randomized controlled trial

**DOI:** 10.1186/s13063-019-3941-z

**Published:** 2019-12-09

**Authors:** Carinne Brody, Sovannary Tuot, Pheak Chhoun, Dallas Swendeman, Kathryn C. Kaplan, Siyan Yi

**Affiliations:** 1 Public Health Program, College of Education and Health Sciences, Touro, University California, Vallejo, CA USA; 2 KHANA Center for Population Health, Research, No. 33, Street 71, Phnom Penh, Cambodia; 3 Department of Psychiatry and Biobehavioral Sciences, University of California, Los Angeles, CA USA

**Correction to: Trials**

**https://doi.org/10.1186/s13063-018-2614-7**

After publication of our article [1] we became aware that several sections of text in our Methods section were copied from a previously published article [2]. We would like to formally apologize and give credit to the authors of that article [2]: Chris Smith, Uk Vannak, Ly Sokhey, Thoai D Ngo, Judy Gold, Khemrin Khut, Phil Edwards, Tung Rathavy and Caroline Free.

Also, one of the author names was misspelled.

Incorrect author name:

- Swendenman

Correct author name:

- Swendeman
