# Supplementary material for: Mobile Link – a theory-based messaging intervention for improving sexual and reproductive health of female entertainment workers in Cambodia: study protocol of a randomized controlled trial
Source: Trials. 2018 Apr 19;19:235. doi: 10.1186/s13063-018-2614-7 (PMC5907699; doi:10.1186/s13063-018-2614-7)
Supplement: Supplementary file 2 — Informed consent form for focus group discussions and in-depth interviews. (DOCX 20 kb) [file 13063_2018_2614_MOESM2_ESM.docx]

**Additional file 2**

**Mobile Link – a theory-based messaging intervention for improving sexual and reproductive health of female entertainment workers in Cambodia: Study protocol of a randomized controlled trial**

**Informed Consent Form for Focus Group Discussions and In-depth Interviews**

My name is XXX. I am an outreach worker with KHANA Center for Population Health Research. I would like to invite you to take part in my research study on ways to deliver health information through text and voice messages to female Entertainment workers. Please read this form carefully and ask any questions you may have before agreeing to take part in the study.

What the study is about: The purpose of this study is to learn how providers and patients feel about the impact of the voucher program on their individual empowerment.

What we will ask you to do: If you agree to be in this study, we will conduct a focus group with you and several other women from the community. The focus group moderator will ask questions about your work, your experience seeking health care and your mobile phone use. You will be asked some questions and can answer and discuss these questions with others in the focus group. The session will last one hour and will be tape recorded. We will transcribe the tape recordings, removing any identifying information such as individual names and then will destroy the tapes. These are standard procedures for focus groups.

Audio-taping: With your permission, I will audiotape and take notes during the focus group. The taping is to accurately record the information you provide. If you choose not to be audio-taped, I will take notes instead. If you agree to being audio-taped but feel uncomfortable at any time during the focus group, I can turn off the tape recorder at your request. Or if you don't wish to continue, you can stop the interview at any time.

Risks and benefits: There are no known discomforts or risks. However, some health issues can be sensitive to discuss in a group. Although we are making every effort to ensure the confidentiality of any information provided, there is a possibility that you could be identified if you provide very specific information on your circumstances. While there are no direct benefits to you, we hope to use the information from you and your colleagues to improve the health care services in the future.

Your answers will be confidential. The records of this study will be kept private. Anything you say in this group in any sort of report we make public we will not include any information that will make it possible to identify you. Research records will be kept in a locked file; only the researchers will have access to the records. To minimize the risks of confidentiality, you will be asked for oral rather than signed consent.

Taking part is voluntary: Taking part in this study is completely voluntary. You may skip any questions that you do not want to answer. If you decide not to take part or to skip some of the questions, it will not affect your current or future relationship with the voucher program. If you decide to take part, you are free to withdraw at any time.

Whether or not you choose to participate in the research and whether or not you choose to answer a question or continue participating in the project, there will be no penalty to you or loss of benefits to which you are otherwise entitled.

We have identified individuals who work in the outreach programs but are not part of the research team to act as witnesses to this process and to sign the consent form once you give your verbal consent.

If you have questions: Please ask any questions you have now. If you have questions later, you may contact XXX.

************************************************************

CONSENT:

If you agree to participate, please say so. You will be given a copy of this form to keep for your own records.

Witness signature:

Date:
