# Supplementary material for: Mobile Link – a theory-based messaging intervention for improving sexual and reproductive health of female entertainment workers in Cambodia: study protocol of a randomized controlled trial
Source: Trials. 2018 Apr 19;19:235. doi: 10.1186/s13063-018-2614-7 (PMC5907699; doi:10.1186/s13063-018-2614-7)
Supplement: Supplementary file 3 — Informed consent for questionnaire survey. (DOCX 21 kb) [file 13063_2018_2614_MOESM3_ESM.docx]

**Additional file 3**

**Mobile Link – a theory-based messaging intervention for improving sexual and reproductive health of female entertainment workers in Cambodia: Study protocol of a randomized controlled trial**

**Informed Consent for Questionnaire Survey**

**Background**

You are being asked to participate in this research study because you work at an entertainment venues that has been selected to participate in a research study about how health related text and voice message may help you achieve your health goals.

We would ask you about your socio-demographic characteristics, relationship, knowledge, attitudes and behaviors related HIV and sexual and reproductive health. It will take approximately 30 minutes.

**Study procedures**

If you agree to participate in this study, you will be asked to participate in three 20-minute surveys that will be administered at baseline, 6-month and 12-months by a trained peer outreach worker from KHANA. They will be recording your information on a tablet. In addition to participating in multiple surveys, you agree to receive at most one SMS/VM per day for one year.

**Risks**

There are no known risks to participation in this study however, some questions are personal in nature and may be uncomfortable to answer. Although we are making every effort to ensure the confidentiality of any information provided, there is a possibility that you could be identified when entering or leaving the private survey location. Additionally, receiving multiple text messages possess a potential risks of confidentiality breach should someone else sees these messages.

**Benefits**

While there are no direct benefits to you for participating in this research study, certain potential benefits may be derived. Such benefits include the possibility that the information learned during this study may help other female entertainment workers in the future. However, none of these potential benefits are guaranteed.

**Voluntary participation**

Your participation in this study is entirely voluntary and you may refuse to participate or discontinue participation at any time without penalty or loss of benefits to which you would normally be entitled. Your decision about whether or not to participate in the study will not affect your relationship with KHANA or any partner organization.

**Alternatives**

Whether or not you choose to participate in the research and whether or not you choose to answer a question or continue participating in the project, there will be no penalty to you or loss of benefits to which you are otherwise entitled.

**Confidentiality**

If you agree to participate in this research, your responses will be used anonymously and shared with others involved in this research but not to the public. For you to be in this research we need your permission to collect and share this information within our research group.

Your protected health information will be kept confidential. Your identity will not be revealed in any publication or presentation of the results of this research.

**Counseling services**

Should you want to speak to someone during or after the research study, you will have access to free counseling services, relevant referrals, escorted referrals and information on Cambodia’s Women’s Crisis Center. You do not need to participate in the research study to access these services.

- Cambodia’s Women’s Crisis Center
- Address: #42F, Str. 488, Phsar Doem Thkauv, Chamkarmon, Phnom Penh, Cambodia.
- Telephone: (855-23) 987158

**Costs and payments**

There are no costs to you for participating in this study. You will be compensated 5,000 riels for your time each time you complete a questionnaire survey.

**Funding Source and Research Study Approval**

We would also like to inform you that this research project is funded by French Government through 5% Initiative. This research project is approved by the National Ethics Committee of the Ministry of Health, Cambodia and Touro University-California Institutional Review Board.

**Questions**

If you have questions or concerns about the research, your research rights, or experience any research related injuries, you may contact the Study Coordinator, Pheak Chhoun at [cpheak@khana.org.kh](mailto:cpheak@khana.org.kh) or the Research Director, Siyan Yi at [ysiyan@khana.org.kh](mailto:ysiyan@khana.org.kh).

You may also contact The National Ethics Committee on Health Research, Dr. Vannat Saray at [sarayvannat@gmail.com](mailto:sarayvannat@gmail.com) or Sarida Nouth at [nouthsarida@gmail.com](mailto:nouthsarida@gmail.com) for any research related questions or concerns.

****************************************************************************

We have identified individuals who work in the outreach programs but are not part of the research team to act as witnesses to this process and to sign the consent form once you give your verbal consent.

Consent to participate in research & authorization to use and share personal health information

I hereby give my verbal consent to participate in this research study and agree that my personal health information can be collected, used and shared by the researchers and staff for the research study described in this form. I will receive a copy of this consent form signed by a witness.

­­­­­­­­­­­­­­­

______________________________________ __________________

Signature of Witness Date
