# Supplementary material for: Mobile Link – a theory-based messaging intervention for improving sexual and reproductive health of female entertainment workers in Cambodia: study protocol of a randomized controlled trial
Source: Trials. 2018 Apr 19;19:235. doi: 10.1186/s13063-018-2614-7 (PMC5907699; doi:10.1186/s13063-018-2614-7)
Supplement: Supplementary file 4 — Focus group discussion and in-depth interview guides. (DOCX 18 kb) [file 13063_2018_2614_MOESM4_ESM.docx]

**Additional file 4**

**Focus Group Discussion Guides**

**Information sheet**

Hello, my name is XXXX. I am an outreach coordinator at KHANA Center for Population Health Research. I would like to invite you to take part in my research study, which is looking at the experiences of women working in the entertainment industry. The purpose of this study is to learn how women feel about receiving health related messages on their phones, what information those message should contain and when you would like to received them.

If you agree to be in this study, we will conduct 2 one-hour focus groups. The focus group will include questions about your experience working in the entertainment industry, your experiences seeking reproductive health care and your thoughts about receiving health related text and voice messages.

The records of this study will be kept private. In any sort of report we make public we will not include any information that will make it possible to identify individual providers. Research records will be kept in a locked file; only the researchers will have access to the records.

Taking part in this study is completely voluntary. If you decide not to take part, it will not affect your current or future relationship with the voucher program. If you decide to take part, you are free to withdraw at any time.

If you would like to proceed, we can set up a time and a place to conduct the interview.

Thank you so much for your consideration.

**Focus Group Discussion Guides**

*Healthy Relationships*

Tell me about the different sexual relationships you or your friends might have in your life (boyfriends, clients, sweethearts, bosses)?

What is the ideal set of relationships? How would you want things *right now* if you could have what you wanted? Monogamous relationship with someone you love? Respectful Clients? Sugar Daddies?

How would you want things *in the future* if you could have what you wanted?

What would your best friend say to you about the relationships in your life right now?

*Health Seeking Behaviors*

What kind of things might prompts you to go to the clinic?

(Friends suggest, client suggests, feel sick/symptoms, notice some change in health, regular check-ups?)

The last time you visit a clinic for your own health, what was that experience like?

What is the ideal experience at a clinic?

What is the ideal relationship with a health provider?

What is something someone you trust might say to you that would encourage you to take really good care of your health? You are worth it? Your family is counting on you?

*Occupational Risks*

At work, what kinds of things make you uncomfortable? Being forced to drink? Men expecting that you will sleep with them? Having your boss harass you?

What would the ideal work environment be like?

What are the condom use norms and rules at the venue where you work? Do they permit condomless sex? Do you get asked?

Are you able to negotiate condom use the way you want?

What things would help make your work environment more like your ideal environment?

**Mobile Link Intervention Development Phase 2 FG**

*HIV Testing*

Tell me about what you know about HIV testing?

What are some reasons someone might not get tested for HIV?

What do you wish someone would tell you about HIV testing?

*Condom Use*

What are some reasons someone might choose not to where a condom with a partner?

Is it hard to talk to clients about condoms?

What do you wish someone would tell you about condoms use?

*Contraception*

What do you think about using modern contraception like the pill or IUD to prevent pregnancy?

What do you wish someone would tell you about contraception?

*STI Testing*

What have you heard about STI testing? When should you get tested?

What do you wish someone would tell you about STI testing?

*Abortion*

Do you think there is a lot of discrimination around abortion in Cambodia?

Do you know where to go if you or a friend wanted a safe abortion?

What do you wish someone would tell you about abortions?

*Violence*

If you were in a situation where you felt threatened physically, what would you do to get to safety?

What do you wish someone would tell you about being free from the threat of violence?

What can someone do who is in a violent relationship and does not feel they can get out?
