# Supplementary material for: Mobile Link – a theory-based messaging intervention for improving sexual and reproductive health of female entertainment workers in Cambodia: study protocol of a randomized controlled trial
Source: Trials. 2018 Apr 19;19:235. doi: 10.1186/s13063-018-2614-7 (PMC5907699; doi:10.1186/s13063-018-2614-7)
Supplement: Supplementary file 5 — Questionnaire for baseline survey. (DOCX 75 kb) [file 13063_2018_2614_MOESM5_ESM.docx]

**Additional file 5**

**Baseline Questionnaire**

For more information, please contact Mr. Pheak Chhoun, Mobile Link Project Coordinator, Research Fellow, KHANA Center for Population Health Research

Mobile: (855) 012 995 866/070 922 888

Landline: (855) 23 211 505 (ext. 390)/Fax: (855) 023 214 049

Address: #33, Street 71, Phnom Penh, Cambodia, PO Box: 2311 Phnom Penh 3

Email: [cpheak@khana.org.kh](mailto:cpheak@khana.org.kh)

**Survey Information:**

| **Variables Name** | **Response** | **Code** |
| --- | --- | --- |
| Study sites | Phnom Penh  Battambang  Banteay Meanchey  Siem Reap | 01  02  03  04 |
| Unique ID code | Study site code+OD code+serial 001 | Eg. 0101001 |
| Date of interview |  |  |
| Interviewer name | A  B  C | 1  2  3 |
| QC name | Sok Ngovlyly |  |

| **Question ID** | **Questions** | **Response** | **Response Code** | **Remark** |
| --- | --- | --- | --- | --- |
| **Section 1: Demographics and background** | | | | |
|  | How old are you at your last birthday? | ............ years |  |  |
|  | In what province were you born? | Province code: ………………… |  |  |
|  | In what type of community were you born? | Rural  Urban | 1  2 |  |
|  | Are your parents both still alive? | No  Yes  Don’t know | 0  1  2 |  |
|  | Thinking of your childhood home, what type of roofing did you have? | Wood planks  Plastic sheet  Metal  Wood  Ceramic tiles  Cement  Not sure  Other:________________ | 1  2  3  4  5  6  7  8 |  |
|  | Thinking of your childhood home, did you have piped water into your home? | No  Yes  Not Sure | 0  1  2 |  |
|  | Thinking of your childhood home, did you have electricity in your home? | No  Yes  Not sure | 0  1  2 |  |
|  | Thinking of your childhood home, what was the main material of the floor? | Natural floor  Wood planks  Ceramic tiles  Floating house  Other:________________ | 1  2  3  4  5 |  |
|  | When you were a child, did you often not have enough food? | No  Yes  Not sure | 0  1  2 |  |
|  | When you were a child, could your family afford to send you to school? | No  Yes | 0  1 |  |
|  | How many years of schooling have you completed? | …………………years  Note: 0 if never attended |  |  |
|  | What is your current marital status? | Married and living together  Married, but not living together  Widowed/divorced/separated  Never married, not living with a sexual partner  Never married, but living with a sexual partner | 1  2  3  4  5 |  |
|  | What type of house are you currently living in? | My own family house  Rental house on my own  Rental house with my family  Rental house shared with friends  Dormitory at my work place  Homeless (on the street or public premise)  Other:________________ | 1  2  3  4  5  6  7 |  |
|  | How many children do you have? | …………………… Children |  |  |
|  | Whom are you currently living with? | Boyfriend/sweetheart  Husband  Family (parents, siblings, children)  Relatives  Friends/colleagues  Other:________________ | 1  2  3  4  5  6 |  |
|  | How many people are dependent on you for living? | ………… people |  |  |
|  | Are there anyone else contributing to support your family? | None  Boyfriend/sweetheart  Husband  Family (parents, siblings, children)  Relatives  Other:________________ | 0  1  2  3  4  5 |  |
| **Section 2: Entertainment work** | | | | |
|  | For how long have you been living in the current city? | ….........Years  0 if not living in this city  1 if living in this city <1 year |  |  |
|  | Have you moved from another city to this city? | No  Yes | 0  1 | 0 Skip to Q21 |
|  | What was the top reason you moved to this city? | For economic opportunities  Following family members  Interest in new places  Getting away from a bad situation  Other:________________ | 1  2  3  4  5 |  |
|  | Have you ever worked in the garment industry? | No  Yes | 0  1 | 0 Skip to Q23 |
|  | Why did you leave the garment industry? | Better pay elsewhere  Better working conditions elsewhere  Was fired or laid off  Interested in a different job  Offered another job  Other:________________ | 1  2  3  4  5  6 |  |
|  | How many months ago did you start working in entertainment industry? | ………………………..months  Note: 1 if less than 1 month |  |  |
|  | What type of venue best describes your first job in entertainment? | Karaoke bar  Massage parlor  Beer garden  Restaurant/cafe  Dance club  Freelance (street, public parks)  Other:________________ | 1  2  3  4  5  6  7 |  |
|  | What type of venue best describes your current job in entertainment? | Karaoke bar  Massage parlor  Beer garden  Restaurant/cafe  Dance club  Freelance (street, public parks)  Other:________________ | 1  2  3  4  5  6  7 |  |
|  | How much money do you typically make per month at your current entertainment job? | ………………………. US$ |  |  |
|  | Are you a part of any organizations that support entertainment/sex workers? | No  Yes | 0  1 |  |
| **Section 3: Sexual behaviors** | | | | |
|  | Have you had sexual intercourse in the past 3 months? | No  Yes | 0  1 | 0 skip to Q44 |
|  | In the past 3 months, did you have sexual intercourse with a partner not in exchange for money or gift (boyfriend/sweetheart)? | No  Yes | 0  1 | 0 skip to Q34 |
|  | In the past 3 months, with how many partners did you have sexual intercourse not in exchange for money or gift? | ………………partner(s) |  |  |
|  | The last time you had sexual intercourse with a partner not in exchange for money or gift, did your partner use a condom? | No  Yes | 0  1 |  |
|  | In the past three months when you had sexual intercourse with a partner not in exchange for money or gifts, how often did you use a condom? | Always  Frequently  Sometimes  Never | 1  2  3  4 |  |
|  | The last time when you had sexual intercourse without a condom with a partner not in exchange for money or gifts, what was the main reason for not using it? | Condom was not available  I requested but my partner did not want to use condom  I requested but my partner convinced me that it was ok  I did not request because I felt uncomfortable or fearful asking  I did not request because I did not think about it  I did not like using condoms  I’m scared that using a condom might have a negative effect on my body  Other:________________ | 1  2  3  4  5  5  6 |  |
|  | In the past 3 months, did you have sexual intercourse with a partner in exchange for money or gift (clients)? | No  Yes | 0  1 | 0 skip to Q44 |
|  | What was the main reason for your involvement in sex work? | In need of money  Suggested by a friend  Lured, cheated or forced into it  To get out of bad family situation  To get out of other work you did not like  Other:________________ | 1  2  3  4  5  6 |  |
|  | How old were you the first time you had sexual intercourse with a partner in exchange for money or gift? | ……………………… years |  |  |
|  | In the past 3 months, how often did you have sexual intercourse with a partner in exchange for money or gifts? | Daily  A few times a week  Weekly  Monthly  Once in a while when I want or need to | 1  2  3  4  5 |  |
|  | Where do you usually meet these types of partners (clients)? | At work  Through friends  At social gatherings  On the street  Advertisement/phone call  Other:________________ | 1  2  3  4  5  6 |  |
|  | In the past 3 months, with how many partners did you have sexual intercourse in exchange for money or gift? | ………………partner(s) |  |  |
|  | In the past 7 days, with how many partners did you have sexual intercourse in exchange for money or gift? | ………………partner(s) |  |  |
|  | The last time you had sexual intercourse with a partner in exchange for money or gift, did your partner use a condom? | No  Yes | 0  1 |  |
|  | In the last 3 months when you had sexual intercourse with a partner in exchange for money or gift, how often did your partners use a condom? | Always  Frequently  Sometimes  Never | 1  2  3  4 |  |
|  | The last time when you had sexual intercourse without a condom with a partner in exchange for money or gift, what was the main reason for not using it? | Condom was not available  I requested but my partner did not want to use condom  I requested but my partner convinced me that it was ok  I did not request because I felt uncomfortable or fearful asking  I did not request because I did not think about it  I did not like using condoms  I’m scared that using a condom might have a negative effect on my body  I was offered more money  Other:________________ | 1  2  3  4  5  6  7  8  9 |  |
| **Section 4: Condom use self-efficacy** | | | | |
|  | Can you discuss condom use with any sexual partner you might have? | Definitely no  Sometimes  Definitely yes | 0  1  2 |  |
|  | Can you insist on condom use whenever you are with someone who says they do not have any disease? | Definitely no  Sometimes  Definitely yes | 0  1  2 |  |
|  | Can you suggest using a condom when you do not initiate the sexual activity? | Definitely no  Sometimes  Definitely yes | 0  1  2 |  |
|  | Can you insist on condom use if a partner does not want to use one? | Definitely no  Sometimes  Definitely yes | 0  1  2 |  |
|  | Can you stop a sexual encounter if a partner insists on not using a condom? | Definitely no  Sometimes  Definitely yes | 0  1  2 |  |
|  | Can you continue to insist on condom use with a person who gets angry when you suggest it? | Definitely no  Sometimes  Definitely yes | 0  1  2 |  |
| **Section 5: HIV risk perception, testing and treatment** | | | | |
|  | Do you feel that you are at risk for HIV? | No  Yes | 0  1 | 0 Skip to Q52 |
|  | IF Yes, why do you feel you are at risk for HIV?  (check all that apply) | I know my partner(s) is HIV +  I frequently have unprotected sex  I do not trust my husband/partner  I shared needles to inject drugs  I have family members who are HIV+  I’m a caretaker for an HIV+ individual  Other____________ | 1  2  3  4  5  6  7 |  |
|  | If No, why don’t you feel you are at risk for HIV?  (Check all that apply) | I only have sex with my husband  I don’t think my partners are HIV+  I don’t feel sick  I always use condoms  I get HIV testing every 3-6 months  I wash after having sex every time  I get cleaned at a clinic frequently  Other_________________ | 1  2  3  4  5  6  7  8 |  |
|  | Have you ever been tested for HIV test? | No  Yes | 0  1 | 0 skip to Q61 |
|  | In the past 6 months, have you been tested for HIV? | No  Yes | 0  1 | 0 skip to Q61 |
|  | If you don’t mind, could you please let us know your HIV status?  Note: You can choose not to answer | HIV(-)  HIV(+)  I don’t know  Don’t want to answer | 0  1  2  3 | 0 skip to Q61 |
|  | Where did you have your most recent HIV test? | Private facilities  Public facilities  NGO facilities  NGO outreach workers at workplace or home  Other_________________ | 1  2  3  4  5 |  |
|  | Are you currently on ART? | No  Yes  I was but discontinued  I am on pre-ART | 0  1  3  4 | 0 skip to Q60 |
|  | Do you take ARV regularly as prescribed? | Not regularly  Regularly 2 | 0  1 |  |
|  | Where do you get ART services? | Public facilities  Private facilities  NGO facilities  Pharmacy  Other_________________ | 0  1  2  3  4 |  |
|  | If not, why are you not on ART? | Not needed  Not sure where to go  Ashamed to go to health facility  Afraid of being discriminated ​​  Badly treated in the past  Other_________________ | 1  2  3  4  5  6 |  |
| **Section 6: STI testing and treatment** | | | | |
|  | In the past 3 months, have you experienced the following symptoms?  [Multiple answers] | No symptoms  Cuts or sores in the genital area  Swelling in the genital area  Abnormal urethral discharge  Symptoms on the anus  Symptom in the mouth/throat | 0  1  2  3  4  5 | 0 skip to Q63 |
|  | Did you seek screening for STI when you had the most recent symptom? | No  Yes | 0  1 | 0 skip to Q66 |
|  | In the past three months, have you been told by a health care provider that you had an STI? | No  Yes | 0  1 | 0 skip to Q66 |
|  | Did you receive treatment for your most recent STI? | No  Yes | 0  1 | 0 skip to Q66 |
|  | Where did you receive the treatment for the most recent STI?  (Only one response) | Pharmacy  Private clinic/hospital  Public clinic/hospital  NGO Clinic/hospital  Traditional healer  Other________________ | 0  1  2  3  4  6 |  |
| **Section 7: Contraceptive and pregnancy** | | | | |
|  | Are you currently using a modern contraceptive to prevent pregnancy? | No  Yes | 0  1 | 0 skip to Q68 |
|  | If yes, what types of contraceptive are you using (check all that apply)? | Condom  Pill  Injectable  Intra-uterus devices (IUD)  Implant (use under the skin)  Female sterilization  Male sterilization  Other________________ | 1  2  3  4  5  6  7  8 |  |
|  | If not, why are you not currently using any modern contraceptive method? | Do not like side effects  Do not think I need contraception  Do not want to prevent pregnancy/want to get pregnant  Do not like using modern methods  Do not know where to get contraception  Do not think I can afford contraception  Other________________ | 1  2  3  4  5  6  7 |  |
|  | Do you usually douche yourself before or after sex as a way to prevent sexually transmitted disease or HIV? | No  Yes | 0  1 | 0 Skip to Q71 |
|  | If yes, how often do you douche yourself after sex as a way to prevent sexually transmitted disease or HIV? | Always  Frequently  Sometimes  Never | 1  2  3  4 |  |
|  | If you wash after sex, what are the main reasons that you wash? | Preventing STI  Preventing HIV  Preventing pregnancy  Stay clean  Avoid smell  Other…………………. | 1  2  3  4  5  6 |  |
|  | Do you think medical abortion (before 12 weeks gestation) is legal in Cambodia? | No  Yes |  |  |
|  | Have you ever experienced unwanted pregnancy? | No  Yes | 0  1 | 0 skip to Q82 |
|  | Have you ever experienced unwanted pregnancy in the past 12 months? | No  Yes | 0  1 |  |
|  | Have you ever had an abortion? | No  Yes |  | 0 skip to Q82 |
|  | During your lifetime, how many abortions have you had? | ………………… times |  |  |
|  | In the past 12 months, how many abortions have you had? | ………………… times |  |  |
|  | The last time you had an abortion, where did you get services? | Pharmacy  Private clinic/hospital  Public clinic/hospital  NGO Clinic/hospital  Traditional healer  Other________________ | 0  1  2  3  4  6 |  |
|  | Did you experience any complication from your most recent abortion such as excessive bleeding or infection? | No  Yes | 0  1 | 0 skip to Q82 |
|  | If yes, did you seek treatment for these complications? | No  Yes | 0  1 | 0 skip to Q82 |
|  | If yes, where did you seek the treatment? | Pharmacy  Private clinic/hospital  Public clinic/hospital  NGO Clinic/hospital  Traditional healer  Other________________ | 0  1  2  3  4  6 |  |
| **Section 8: Gender-based violence** | | | | |
|  | Have you experienced unwanted touching or groping in the past 3 months? (at work?) | No  Yes  Don’t Know | 0  1  2 |  |
|  | What can you do if you or a female friend or family member experience physical or sexual abuse? | There is nothing to do  Go to local authorities  Go to police or courts  Tell other family and friends  Go to an NGO  Other________________ | 1  2  3  4  5  6 |  |
|  | If a wife does not obey a husband, do you think he is justified in hitting, kicking or beating her? | No  Yes  Sometimes | 0  1  2 |  |
|  | If a wife does not obey a husband, do you think he is justified in yelling at her? | No  Yes  Sometimes | 0  1  2 |  |
|  | If a girlfriend does not obey a boyfriend, do you think he is justified in hitting, kicking or beating her? | No  Yes  Sometimes | 0  1  2 |  |
|  | Do you think it is a woman’s duty to have sex with her husband, even when she does not want to? | No  Yes  Sometimes | 0  1  3 |  |
|  | What type of violence, if any, have you ever experienced in your lifetime?  (check all that apply)? | Verbal threats  Controlling ability to leave house  Hit, slapped, kicked or beat you?  Forced sex  Other________________  None | 1  2  3  4  5  6 |  |
|  | What type of violence, if any, have you experienced in the past six months (check all that apply)? | None  Verbal threats  Controlling ability to leave house  Hit, slapped, kicked or beat you  Forced sex  Other________________ | 1  2  3  4  5  6 |  |
|  | Who was the **main** perpetrator (s) of violence *check all that apply* | Family member  Friend  Husband/Partner  Sweetheart  Manager  Client  Co-worker  Stranger  Police  Moto/taxi driver  Other *please specify* | 1  2  3  4  5  6  7  8  9  10  11 |  |
| **Section 9: Substance abuse** | | | | |
|  | In the past 3 months, how often did you drink at least one can of beer or one glass of any types of wine?  - A glass of wine (120 ml)  - A glass of whisky (30 ml) | Never  Once a month or less  2-4 times a month  2-3 times a week  4 or more times a week | 0  1  2  3  4 | 0 skip to Q94 |
|  | In the past 3 months, how many standard drinks containing alcohol (a can of beer or a glass of any types of wine) did you have on a typical day on which you drank alcohol? | 1 - 2  3 – 4  5 - 6  7 - 9  10 or more | 0  1  2  3  4  5 |  |
|  | In the past 3 months, how often did you have more than 5 drinks in one day or night? | Never  Less than once a month  Once a month  1, 2, or 3 times a week  4 or more times a week | 0  1  2  3  4  5 |  |
|  | In the past 3 months, how often have you been forced to drink alcohol at work when you did not want to drink? | Never  Less than once a month  Once a month  1-3 times/week)  4 or more times a week | 0  1  2  3  4  5 |  |
|  | In the past 3 months, have you used any of the following drugs?  (Ask one by one – CIRCLE YES OR NO)   \|  \| Types of drugs \| Yes \| No \| \| --- \| --- \| --- \| --- \| \| 1 \| Marijuana \| 1 \| 0 \| \| 2 \| Heroin/opium \| 1 \| 0 \| \| 3 \| Yama (amphetamine) \| 1 \| 0 \| \| 4 \| Crystal, Ice (methamphetamine) \| 1 \| 0 \| \| 5 \| Ecstasy \| 1 \| 0 \| \| 6 \| Inhalants (glue, paint, petrol, spray can) \| 1 \| 0 \| \| 7 \| Other \| 1 \| 0 \| | | | |
|  | In the past 3 months, have you injected any illicit drugs? | Never injected any drug  Heroin  Yama  Crystal, Ice  Other________________ | 0  1  2  3  4 |  |
|  | In the past 3 months, have you had sex during or after using illicit drugs? | Never  Heroin  Yama  Crystal, Ice  Other________________ | 0  1  2  3  4 |  |
| **Section 10: Linkage to services** | | | | |
|  | Have you ever contacted an outreach worker to ask a health question in the past 6 months? | No  Yes | 0  1 | 0 end of interview |
|  | What health issues did you contact her about?  (check all that apply) | HIV  STIs  Family planning  Gender-based violence  General emotional support  Vaginal health (discharge, irritation, inflammation)  Legal support  Other________________ | 1  2  3  4  5  6  7  8 |  |
|  | In the past 6 months, how many times have you contacted an outreach worker? | 1 time  2-4 times  5+ times | 1  2  3 |  |
|  | Have you ever received an escorted referral for a health services from an outreach worker? | No  Yes | 0  1 |  |
|  | For what health issues did you receive an escorted referral?  (check all that apply) | HIV  STIs  Family planning  Gender-based violence  General emotional support  Vaginal health (discharge, irritation, inflammation)  Legal support  Other________________ | 1  2  3  4  5  6  7  8 |  |

Thank you for your participation!
